# Characterization of Pana g 1, an important cause of pollen-food allergy syndrome from Korean ginseng, *Panax ginseng*^☆^

**DOI:** 10.1016/j.waojou.2025.101164

**Published:** 2026-01-03

**Authors:** Kyoung Yong Jeong, Yoon Ji Shin, Haeun Kim, Yong Seok Lee, Minkyu Sang, Hyun Kyung Oh, Kyung Hee Park, Jae-Hyun Lee, Jung-Won Park

**Affiliations:** a Department of Internal Medicine, Institute of Allergy, Yonsei University College of Medicine, Seoul, South Korea; b Department of Biology, Soonchunhyang University, Asan, South Korea

**Keywords:** Ginseng, Pathogenesis-related 10 protein, Pollen-food allergy syndrome

## Abstract

**Background:**

Ginseng is a widely consumed herbal supplement. However, ginseng, especially raw ginseng, can cause allergic reactions, including pollen-food allergy syndrome (PFAS). This study aimed to identify the PFAS-causative allergen in Korean ginseng and to establish methods for its quantification.

**Methods:**

Candidate allergens were screened using genomic and transcriptomic analyses. Proteomic profiling with patient sera was performed to identify the clinically relevant allergen. A recombinant protein was generated, and its allergenicity compared with the primary sensitizer, Que ac 1, by ELISA. A two-site ELISA was developed for the quantification of the ginseng allergen using monoclonal antibodies against recombinant protein. Multiple reaction monitoring (MRM) mass spectrometry was applied for validation.

**Results:**

Genome and transcriptome analysis identified 4 candidate allergens: pathogenesis-related 10 (PR-10) protein, profilin, non-specific lipid transfer protein (nsLTP), and thaumatin-like protein. Among these, PR-10 (designated Pana g 1) was the sole allergen detected by proteomic analysis. Recombinant Pana g 1 was recognized by 4 of 5 patients. Inhibition ELISA showed stronger IgE reactivity to Que ac 1 than to Pana g 1, with marked cross-reactivity between the 2. Pana g 1 levels in ginseng extract were quantified as 4.26 μg/mg of protein by ELISA and 4.54 μg/mg by MRM in the ginseng extract.

**Conclusion:**

Pana g 1 is the major PFAS-causative allergen in Korean ginseng. Recombinant Pana g 1 shows promise as a diagnostic tool for ginseng-induced PFAS. The quantification systems established here may also support standardization of ginseng extracts and allergen monitoring.

## Introduction

Korean ginseng (*Panax ginseng* Meyer) is widely utilized as an herbal supplement in Chinese and Korean traditional medicine for its anti-inflammatory properties, mainly attributed to ginsenosides, a class of saponins.^1^ However, randomized controlled trials have not substantiated substantial medical benefits,^2^ and data on adverse reactions associated with ginseng abuse or misuse remain limited.^3^

Pollen-food allergy syndrome (PFAS) is an IgE-mediated condition occurring in pollen-sensitized individuals after ingestion of raw fruits and vegetables due to allergen cross-reactivity. Raw ginseng is recognized as a frequent PFAS elicitor in Asia, where consumption of raw honey-marinated ginseng is common, particularly in Korea. In a nationwide survey of 648 pollinosis patients, ginseng accounted for 8.2% of PFAS cases.^4^ In another study of 273 PFAS, including 27 with anaphylaxis, ginseng was implicated in 14.8% of cases, making it a notable cause of severe reactions.^5^ Anaphylaxis linked to ginseng PFAS has been confirmed by oral challenge and basophil activation tests,^6^ and an additional case was reported after intravenous administration of Shenmai injection, a traditional Chinese medicine preparation containing ginseng as a principal ingredient.^7^ Moreover, inhalation of dried ginseng powder has been associated with occupational asthma among traditional medicine practitioners.^8^

Pathogenesis-related protein 10 (PR-10) from ginseng has long been suspected as a causative allergen in PFAS. However, its allergenic properties remain poorly defined, despite the recent availability of draft genome sequences for ginseng.^9^ In Korea, oak species are the dominant sensitizers in spring pollinosis, accounting for approximately 50% of deciduous trees, whereas birch species represent only about 2% (Forest statistical system: https://kfss.forest.go.kr/stat/ptl/fyb/frstyYrBookList.do?curMenu=9854). Strong IgE cross-reactivity between these 2 taxa has been well documented.^10^ Accordingly, cross-reactivity between ginseng PR-10 and Que ac 1, a major allergen from sawtooth oak (a primary sensitizer in spring pollinosis), has been a focus of investigation.^11^^,^^12^ However, it should also be noted that oak is not a single sensitizer even in Korea; individual history can vary, and co-sensitization to homologous pollen, including birch, is also common.

In this study, we characterized the allergenicity of ginseng PR-10 and established quantification methods for its content. These included monoclonal antibodies (mAbs) raised against recombinant ginseng PR-10 and multiple reaction monitoring (MRM)-MS/MS spectrometry. The ginseng PR-10 allergen has now been designated ‘Pana g 1’ and listed in the WHO/IUIS Allergen Nomenclature Database.

## Methods

### Serum samples

Serum samples were obtained from 5 allergy patients with a history of ginseng-induced allergy (average age 40 years, range 21–68 years) who visited the Allergy-Asthma Center at Severance Hospital, Seoul, Korea (Table 1). All patients were diagnosed with pollen-food allergy syndrome (PFAS) following ginseng ingestion, based on the immediate allergic reactions confirmed by allergists. Allergn-specific IgE was measured using the ImmunoCAP (ThermoFisher Scientific, Uppsala, Sweden). A positive skin prick test response was defined as a wheal size ≥3 mm, or erythema ≥10 mm, or allergen-to-histamine ratio ≥1. Written informed consent was obtained from all participants prior to blood collection. This study protocol was approved by the Institutional Review Board of Yonsei University Health System (IRB no. 4-2022-0733).

**Table 1:** Clinical features of the enrolled patients.

No.	Gender	Age	Diagnosis^a^	Sensitization profile (sIgE, kU_A_/L measured by ImmunoCAP)^b^	Clinical history
1	F	55	AR, PFAS	t17 (4.58), t215 (0.04), w1 (25.9), w6 (20.7), w8 (14.5), w12 (7.50)	celery, chicory, ginseng, radish sprouts, toothed ixeridium
2	F	51	AR, PFAS	t215 (0.28), w6 (22.3) SPT: Histamine (6x6/15x15), d1 (3x3/5x5), d2 (3x3/4x4), e1 (5x5/20x20), e3 (5x5/30x20), e5 (5x5/25x20), w1 (5x5/10x8), w6 (20x12/35x25), w8 (8x8/20x15), w10 (5x5/15x10),	fresh ginseng, mango
3	F	27	AR, PFAS	f31 (0.42), f299 (0.97), t215 (21.3)	ginseng, jujube, uncooked chestnut
4	F	68	AR, PFAS	f31 (5.13), f85 (9.51), f299 (1.55), t215 (56.6) SPT: Histamine (4x4/10x10), f12 (4x3/20x15), f13 (3x3/12x12), f17 (3x3/15x15), f20 (3x3/15x3), f85 (4x3/15x12), sesame (3x3/10x10)	carrot, celery, deodeok, ginseng, uncooked chestnut,
5	M	21	AA, AR, CSU, PFAS	d2 (0.61), e1 (19.6), e5 (1.25), f49 (10.7), f95 (18.2), f255 (0.41), m6 (1.00), t3 (36.6), t7 (30.4) SPT: Histamine (4x3/20x17), e1 (5x5/22x20), f12 (4x3/22x20), f14 (6x4/30x25), f93 (3x3/25x20), t3 (20x12/40x30), t7 (9x5/25x20)	apple, cherry, jujube, peach, pineapple, plum, soy milk, uncooked chestnut, wild ginseng,

Positive SPT: wheal size ≥3 mm or erythema ≥10 mm or allergen/histamine ratio ≥1, all SPT negative control = 0, histamine or allergen (wheal size/erythema size).

a AA, allergic asthma; AR, allergic rhinitis; CSU, chronic spontaneous urticaria; PFAS, pollen food allergy syndrome; SPT, skin prick test.

b d1, *D. pteronyssinus*; d2, *D. farinae*; e1, cat dander; e3, horse hair; e5, dog hair; f4, wheat; f12, peas; f13, peanut; f14, soybean whole; f17, hazelnut; f20, almond; f31, carrot; f49, apple; f85, celery; f93, cacao; f95, peach; f255, plum; f301, persimmon; f299, sweet chestnut; m6, *Alternaria alternata*; t3, birch; t7, oak; t17, Japanese cedar; t215, Bet v 1; w1, ragweed; w6, mugwort; w8, dandelion; w10, Chenopodium; w12, golden rod

### Preparation of oak pollen and ginseng extract

Fresh ginseng was sourced from a local market. The roots were homogenized using a Waring blender and extracted in phosphate-buffered saline (PBS, pH 7.4) at a 1:3 (w/v) ratio for 24 h at 4 °C. The extract was centrifuged at 15,000 rpm (≈27,000×*g*) for 30 min at 4 °C, and the supernatant was dialyzed against distilled water using a 10 kDa cutoff membrane. Sawtooth oak pollen extract was obtained from Prolagen Ltd. (Seoul, Korea). Protein concentration was determined by the Bradford assay. All extracts were aliquoted and stored at −70 °C until use.

### Transcriptome and genome analysis

Potential allergen homologues were identified using the ginseng sequence read archive (SRA) and genome database (GCA_020205605.1). Query sequences included Bet v 1, 2, 3, 4, 6, 7, 8, and thaumatin-like protein. Homology searches were performed with BLAST (BLASTX for protein sequences and BLASTN for nucleotide sequences) using an E-value cutoff of 1 × 10^−5^.^13^ Gene ontology (GO) analysis was conducted based on PANM DB annotation with professional software BLAST2GO (http://www.BLAST2go.org/). Unigenes were classified into level 2 categories using WEGO software (http://wego.genomics.org.cn/cgi-bin/wego/index.pl/).^14^

### Identification of ginseng allergen by proteome analysis

Allergen extracts from Korean ginseng and sawtooth oak (20 μg/well each) were separated by 15% SDS-PAGE and electroblotted onto a nitrocellulose membrane. Membranes were blocked with 3% skim milk in phosphate buffered saline containing 0.5% Tween 20 (PBST) and incubated overnight with pooled patient sera (1:4 dilution; from 2 patients positive for recombinant protein). IgE reactivity was probed with pooled sera, and bound IgE antibodies were detected with alkaline phosphatase-conjugated goat anti-human IgE (1:1000) (Sigma-Aldrich, St. Louis, MO, USA) for 1 h. Color development was achieved with nitro blue tetrazolium and 5-bromo-4-chloro-3-indolyl-phosphate (NBT/BCIP; Promega, Madison, WI, USA).

For the identification of IgE-reactive components, 2-dimensional gel electrophoresis followed by IgE immunoblotting was performed, and protein spots were analyzed by liquid chromatography-coupled electrospray ionization tandem mass spectrometry (LC-coupled ESI MS/MS) analysis.

### Recombinant protein expression and purification

Complementary DNA (cDNA) was synthesized and ligated into the pBT7-*N*-His expression vector (Bioneer, Daejeon, Korea), which was transformed into *Escherichia coli* Rosetta-gami 2 cells. Recombinant protein expression was induced with 1 mM isopropyl-1-thio-β-galactopyranoside (IPTG). The expressed protein was isolated from inclusion bodies using Ni-affinity chromatography. Production of recombinant Que ac 1 was described in a previous study.^11^

### ELISA for IgE reactivity

Recombinant Pana g 1 and Que ac 1 (2 μg/mL) were coated onto polystyrene microplates and incubated overnight at 4 °C, followed by blocking with 3% skim milk for 1 h at room temperature. Serum samples from ginseng-allergic patients and healthy controls, diluted 1:4 in PBST containing 1% BSA, were added to the microplates and incubated for 1 h. Bound IgE was detected using biotinylated goat anti-human IgE antibodies (1:1000; Vector, Burlingame, CA, USA) for 1 h, followed by streptavidin-peroxidase conjugate (1:1000) (Sigma-Aldrich) for 30 min. Color development was achieved with 3,3′5,5′-tetramethyl-benzidine (TMB) substrate (SeraCare Life Sciences, Milford, MA, USA), and the reaction was stopped with 0.5 M H_2_SO_4_. Absorbance was measured at 450 nm using a microplate reader. The cutoff for positivity was defined as the mean absorbance plus 2 standard deviations of healthy control sera.

### Inhibition ELISA for cross-reactivity

Cross-reactivity between Pana g 1 and Que ac 1 was evaluated by inhibition ELISA. The assay conditions were identical to those described above, except for serum preparation. Pooled sera were pre-incubated overnight with recombinant Pana g 1 and Que ac 1 (0, 0.0032, 0.016, 0.08, 0,4, 2, 10 μg/mL) prior to being added to microplates coated with the respective recombinant proteins. The degree of inhibition was calculated using the formula: Inhibition (%) (1-A_i_/A_0_) × 100, where A_i_ represents absorbance with an inhibitor and A_0_ represents absorbance without an inhibitor.

### Quantification of Pana g 1 content by mass spectrometry

Five target peptides (TEVEATSTVPAQK, LYAGLLLDIDDILPK, AFPQAIK, SSEIIEGDGGVGTVK, LVTLGEASQFNTMK) were selected using Skyline software (www.skyline.ms). Synthetic peptides (>97% purity) were generated, and unscheduled MRM was performed on an Agilent 6495 (QqQ) mass spectrometer coupled with an Agilent 1290 (LC) system. Each peptide (200 fmol) was analyzed to evaluate detectability, including peptide elution time and peak abundance. Quantification of Pana g 1 in ginseng extract was achieved by comparison with internal standards.

A calibration curve was plotted using 12 different concentrations of the peptides (10, 25, 50, 100, 250, 500, 1000, 1250, 5000, 10000, and 20000 fmol), with triplicate analyses performed. Only results with an r^2^ above 0.99 were accepted for further analysis. The coefficient of variation (CV), limit of detection (LOD), and limit of quantification (LOQ) were determined to assess linearity and reproducibility. LOD was calculated as 3.3 × σ/s, where σ represents the standard deviation (SD) of peak area from blank analysis and s denotes the gradient of the calibration curve. LOQ was defined as 10 × σ/s^2^.

For sample preparation, acetone precipitation of the ginseng extract was used to concentrate and remove salts. Peptides were desalted following trypsin digestion using an OASIS-HLB 6 cc Vac cartridge (Waters, Milford, MA, USA).

### Development of mAbs for Quantification of Pana g 1 content

Mouse mAbs were generated by fusing splenocytes from BALB/c mice immunized with recombinant Pana g 1 to myeloma cells (Sp 2.0-Ag 14). Mice were immunized 3 times at two-week intervals with recombinant protein emulsified in Freund's adjuvants. Hybridomas secreting antibodies against recombinant Pana g 1 were screened by ELISA and western blotting using ginseng extract. Four positive hybridoma clones were expanded through limiting dilution, and mAbs were purified using protein G affinity chromatography (Sigma-Aldrich, St. Louis, MO, USA). Antibody isotypes were determined using a commercial isotyping reagents (Pierce, ThermoFisher Scientific, Waltham, MA, USA).

### Biotinylation of antibodies

Purified monoclonal antibodies were biotinylated with EZ-link® Sulfo–NHS–LC-Biotin (ThermoFisher Scientific). Briefly, antibodies (1 mg/mL) were incubated with NHS-LC-Biotin on ice for 4 h, followed by extensive dialysis against PBS to remove unreacted reagent.

### Two-site ELISA for pana g 1 quantification

The optimal antibody pair for Pana g 1 detection was determined by comparing titration curves obtained with 4 monoclonal antibodies. The combination of biotinylated 22C3 (1:1000 dilution) as the detection antibody and 15B12 (10 μg/mL) as the capture antibody provided the best results. The capture antibody (15B12, 10 μg/mL in carbonate buffer, pH 9.6) was coated onto microplates overnight at 4 °C. Plates were blocked with 3% skim milk, and serial dilutions of antigens (ginseng extract or recombinant Pana g 1) were added and incubated for 1 h at room temperature. The detection antibody (biotinylated 22C3, 1:1000 dilution) was then applied for 1 h, followed by, streptavidin-conjugated peroxidase (1:1000; Sigma-Aldrich) for 30 min. Plates were washed at least 3 times with PBST between steps. Color was developed using TMB Microwell Peroxidase Substrate (SeraCare Life Science, Inc., Milford, MA, USA), and the reaction was terminated with 0.5 M H_2_SO_4_. Absorbance was measured at 450 nm using a microplate reader. All samples were analyzed in duplicate.

In addition, Pana g 1 content was quantified in a range of commercial products, including red ginseng extract sticks, ginseng tea, and beverages containing red ginseng.

### Recognition of pana g 1 homologues by mAbs

Immunoblotting was performed to evaluate whether the mAbs recognize homologous molecules in apple (DST Diagnostische Systeme & Technologien GmbH, Schwerin, Germany), peanut (Prolagen Ltd, Seoul, Korea), and sawtooth oak pollen extracts (Prolagen Ltd). Protein extracts were separated on 15% SDS-PAGE and either stained with Coomassie blue or electroblotted onto PVDF membranes. Membranes were blocked with 3% skim milk in PBST and incubated for 1 h with hybridoma culture supernatants (15B12 and 22C3). Bound antibodies were detected using alkaline phosphate-conjugated goat anti-mouse IgG (Fab-specific, Sigma-Aldrich) (1:1000 dilution) and visualized using NBT and BCIP substrate.

## Results

### Identification and cloning of pana g 1

**Fig. 1:**
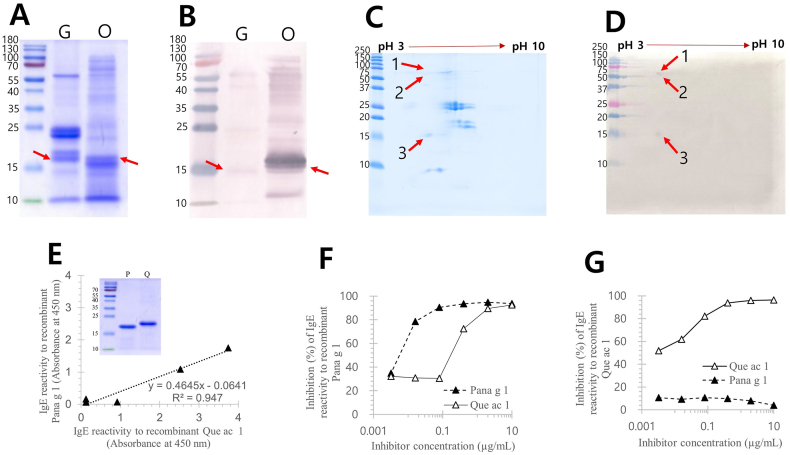
Identification and IgE reactivity of recombinant Pana g 1. Protein profile of ginseng extract and sawtooth oak pollen extract shown by SDS-PAGE (A). IgE reactive component from ginseng and sawtooth oak extract (B). Two-dimensional gel analysis of ginseng extract (C). IgE reactive components of ginseng extract in 2D gel (D). Comparison of IgE reactivity to Pana g 1 and Que ac 1 by ELISA (E). Inset: purified recombinant allergens. ELISA inhibition of IgE binding by recombinant Pana g 1 and recombinant Que ac 1 against recombinant Pana g 1 (F) and Que ac 1 (G). G, ginseng extract; O, sawtooth oak pollen extract; P, Pana g 1; Q, Que ac 1. Arrows indicate Pana g 1 and Que ac 1.

**Fig. 2:**
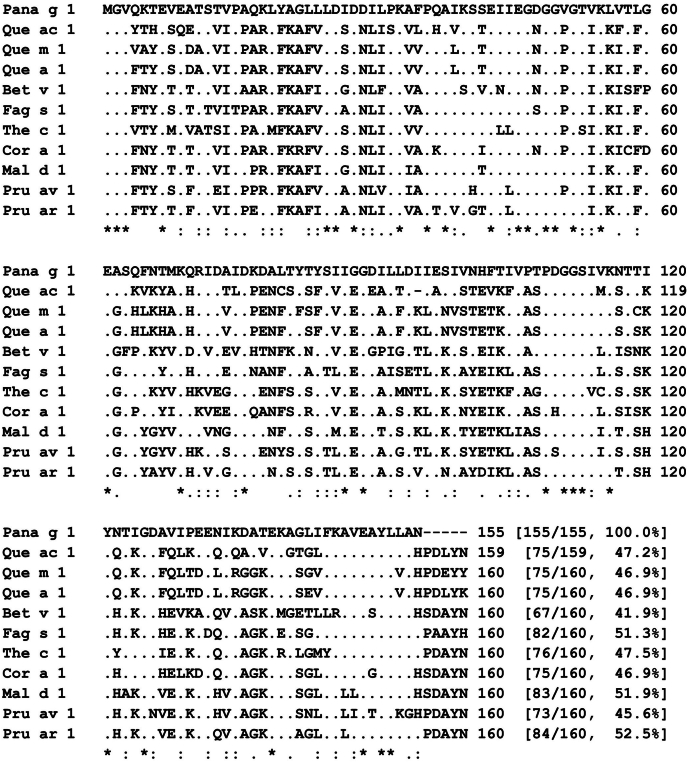
Sequence alignment of Pana g 1 with PR-10 allergens. Pana g 1.0101 (*Panax ginseng*, Korean ginseng, ADW93867.1); Que ac 1.0101 (*Quercus acutissima*, sawtooth oak, QOL10866); Que m 1.0101 (*Q. mongolica*, Mongolian oak, AUH28179); Que a 1.0101 (*Q. alba*, white oak, ABZ81046); Bet v 1.0101 (*Betula verrucosa*, European white birch, CAB02154); Fag s 1.0101 (*Fagus sylvatica*, European beech, ACJ23864); cacao PR-10 (*Theobroma cacao*, cacao, XP_007035697); Cor a 1.0301 (*Corylus avellana*, European hazelnut, CAA96549); Mal d 1.0401 (*Malus domestica*, apple, CAA96535); Pru av 1.0101 (*Prunus avium*, sweet cherry, AAC02632); Pru ar 1.0101 (*Prunus armeniaca*, apricot, AAB97141).

SDS-PAGE and IgE immunoblot analysis indicated that IgE reactivity to oak was markedly stronger than to ginseng extract (Fig. 1A and B), despite the fact that the quantified content of PR-10 was more than 2.5-fold higher in ginseng extract compared with oak, determined by 2 different ELISA systems (4.3 μg/mL of Pana g 1; 1.7 μg/mL of Que ac 1). IgE binding to ginseng extract targeted proteins of approximately 15, 25, 45, and 60 kDa, whereas oak reactivity to oak was largely restricted to a single 17 kDa component. The proteomic profiling of the ginseng extract identified 3 IgE-reactive spots (Fig. 1C and D), but LC-coupled ESI MS/MS analysis confirmed that only 1 allergenic protein, Pana g 1, a member of the PR-10 family (Table 2). Sequence analysis further revealed that Pana g 1 shared 41.9%–52.5% sequence identity with other PR-10 allergens (Fig. 2).

**Table 2:** Identification of ginseng allergens by mass spectrometry

Spot label	No.	Accession No.	Protein name	Mascot Score	Mass (Da)/pI	Expect	Diagnostic peptide
1 (65 kDa, pI 4.3)	1	RWR58290.1	Multidrug resistance efflux transporter family protein [*Bacillus cereus*]	30	34,541/8.36	0.019	R.AMDLEGGSWIWSASLR.Y + Oxidation (M)
	2	AOH84647.1	Ribosome biogenesis GTPase Der [*Sphingomonas panacis*]	30	49,035/5.60	0.023	R.IADAALQEGR.A
	3	RIZ42146.1	Succinylglutamate desuccinylase [*Pseudomonas putida*]	27	41,181/5.83	0.047	R.LAELEQQGR.L
2 (55 kDa, pI 4.2)	1	RWR55878.1	50S ribosomal protein L5 [*Bacillus cereus*]	27	20,153/9.70	0.039	K.LVSVSLPR.V
3 (15 kDa, pI 4.6)	1	ADW93869.1	Pathogenesis related 10-2 [*Panax ginseng*]	1567	16,613/4.58	0.0055 2.9e-006 0.00024 0.0018 0.00074	K.GSFLDMDTVVPK.A + Oxidation (M) K.SVQVLEGNGGVGTIK.N + Deamidated (NQ) K.NVTLGDATPFNTMK.T + Oxidation (M) K.SVQVLEGNGGVGTIKNVTLGDATPFNTMK.T + Deamidated (NQ); Oxidation (M) K.IVPTDGGSTITQTTIYNTIGDAVIPEENVKDATEK.S
	2	ACY36943.1	Pathogensis-related protein 10 [*Panax ginseng*]	1551	16,613/4.56	0.0055 2.9e-006 0.00024 0.0018 0.001	K.GSFLDMDTVVPK.A + Oxidation (M) K.SVQVLEGNGGVGTIK.N + Deamidated (NQ) K.NVTLGDATPFNTMK.T + Oxidation (M) K.SVQVLEGNGGVGTIKNVTLGDATPFNTMK.T + Deamidated (NQ); Oxidation (M) K.IVPTDGGSTITQTTIYNTIGDAVIPEENIKDATDK.S
	3	sp|P80889.1|RNS1_PANGI	Ribonuclease 1	616	16,414/4.41	3.0e-005 5.6e-005 0.012	K.SSEIIEGDGGVGTVK.L K.LVTLGEASQFNTMK.Q + Oxidation (M) K.LYAGLLLDIDDILPK.A
	4	PMS26606.1	2-Aminoethylphosphonate ABC transport system ATP-binding subunit PhnT [*Trinickia soli*]	58	39,539/6.86	0.0047	R.TVLDDLSLSIAR.G
	5	PMS28201.1	ABC transporter [*Trinickia soli*]	29	65,287/7.64	0.031	R.VVAGLVER.A

### Cloning, expression, and allergenicity of Recombinant Pana g 1

Ginseng molecules homologous to previously described allergens were identified through transcriptomic and genomic analyses (Table 3). Among 5 PR-10 sequences deposited in the NCBI protein database, we selected the sequence most similar to Que ac 1, a major allergen responsible for spring pollinosis in Korea. Sequence comparison revealed that the PR-10 protein shares 41.9% identity with Bet v 1 and up to 52.5% with Pru ar 1.

**Table 3:** Allergen homologues obtained by the analyses of transcriptome and genome

Biochemical identity	Allergen	Species	Sequence identity	Subject ID
Pathogenesis related 10 protein	Que ac 1 Que m 1 Que a 1 Bet v 1 Fag s 1 The c 1 Cor a 1 Mal d 1 Pru av 1 Pru ar 1	Sawtooth oak (*Quercus acutissima*) Mongolian oak (*Quercus mongolica*) White oak (*Quercus alba*) European white birch (*Betula verrucosa*) European beech (*Fagus sylvatica*) Cacao (*Theobroma cacao*) European hazelnut (*Corylus avellana*) Apple (*Malus domestica*) Sweet cherry (*Prunus avium*) Apricot (*Prunus armeniaca*)	47.2% 46.9% 46.9% 41.9% 51.3% 47.5% 46.9% 51.9% 45.6% 52.5%	CM035149.1
Profilin	Aca f 2 Ama r 2 Amb a 8 Ana c 1 Api g 4 Ara h 5 Art v 4 Bet v 2 Can s 2 Cap a 2 Che a 2 Citr l 2 Cit s 2 Cor a 2 Cuc m 2 Cyn d 12 Dau c 4 Fra a 4 Hev b 8 Jug r 7 Mal d 4 Mus a 1 Pru du 4 Pru p 4 Pyr c 4 Que ac 2 Tri a 12 Zea m 12	Needle bush (*Vachellia farnesiana*) Redroot pigweed (*Amaranthus retroflexus*) Short ragweed (*Ambrosia artemisiifolia*) Pineapple (*Ananas comosus*) Celery (*Apium graveolens*) Peanut (*Arachis hypogaea*) Mugwort (*Artemisia vulgaris*) European white birch (*Betula pendula*) Indian hemp (*Cannabis sativa*) Bell pepper (*Capsicum annuum*) White goosefoot (*Chenopodium album*) Watermelon (*Citrullus lanatus*) Sweet orange (*Citrus sinensis*) Hazelnut (*Corylus avellana*) Muskmelon (*Cucumis melo*) Bermuda grass (*Cynodon dactylon*) Carrot (*Daucus carota*) Strawberry (*Fragaria vesca* subsp. *vesca*) Para rubber tree, latex (*Hevea brasiliensis*) English walnut (*Juglans regia*) Apple (*Malus domestica*) Banana (*Musa acuminate*) Almond (*Prunus dulcis*) Peach (*Prunus persica*) Pear (*Pyrus communis*) Sawtooth oak (*Quercus acutissima*) Wheat (*Triticum aestivum*) Maize (*Zea mays*)	70.7% 72.9% 68.4% 77.1% 75.4% 79.4% 68.4% 72.2% 72.9% 83.2% 78.6% 77.1% 72.5% 78.6% 75.6% 76.3% 75.6% 80.2% 77.1% 73.7% 74.8% 77.9% 74.8% 74.8% 75.4% 73.7% 71.8% 75.6%	CM035138.1
Nonspecific lipid transfer protein	Act c 10 Act d 10 Fra a 3 Hel a 3 Hev b 12 Jug r 3 Mal d 3 Mor n 3 Pis s 3 Pru ar 3 Pru av 3 Pru d 3 Pru du 3 Pru p 3 Pun g 1 Rub i 3 Sola l 1 Vit v 1 Art ca 3 Art an 3 Art ar 3 Art si 3 Art gm 3 Art la 3 Pla or 3	Gold kiwi fruit (*Actinidia chinensis*) Green kiwi fruit (*Actinidia deliciosa*) Strawberry (*Fragaria ananassa*) Sunflower (*Helianthus annuus*) Para rubber tree, latex (*Hevea brasiliensis*) English walnut (*Juglans regia*) Apple (*Malus domestica*) Mulberry (*Morus nigra*) Pea (*Pisum sativum*) Apricot (*Prunus armeniaca*) Sweet cherry (*Prunus avium*) European plum (*Prunus domestica*) Almond (*Prunus dulcis*) Peach (*Prunus persica*) Pomegranate (*Punica granatum*) Red raspberry (*Rubus idaeus*) Tomato (*Solanum lycopersicum*) Grape (*Vitis vinifera*) Wormwood (*Artemisia capillaris*) Sweet wormwood (*Artemisia annua*) Silvery wormwood (*Artemisia argyi*) Sieversian wormwood (*Artemisia sieversiana*) Russian wormwood (*Artemisia gmelinii*) Mugwort (*Artemisia lavandulifolia*) Oriental plane tree (*Platanus orientalis*)	45.6% 52.2% 42.7% 33.6% 44.8% 41.2% 41.7% 56.0% 44.2% 48.4% 42.7% 46.2% 52.9% 49.5% 41.7% 41.0% 48.3% 50.0% 38.5% 38.5% 40.5% 37.9% 38.5% 40.5% 45.8%	CM035130.1
Thaumatin-like protein	Cup s 3 Jun a 3 Ole e 13 Act d 2 Mal d 2 Pru p 2 Pru av 2 Mus a 4 Cap a 1	Common cypress (*Cupressus sempervirens*) Mountain cedar (*Juniperus ashei*) Olive (*Olea europaea*) Green kiwi (*Actinidia deliciosa*) Apple (*Malus domestica*) Peach (*Prunus persica*) Sweet cherry (*Prunus avium*) Banana (*Musa acuminata*) Chilli or bell pepper (*Capsicum annuum*)	48.0% 48.9% 62.0% 71.1% 33.1% 34.6% 36.3% 70.0% 58.1%	CM035148.1

To produce recombinant protein, cDNA encoding Pana g 1 - the sole allergen identified by proteomic analysis - was synthesized in a pBT7-*N*-His vector. Following Ni-affinity chromatography purification, recombinant Pana g 1 exhibited the expected size of 19.287 kDa, including the *N*-terminal tagging sequence (MHHHHHHSSGLVPRGSEFSQQDSD-), with a yield of 11.195 mg/L of bacterial culture.

IgE reactivity to recombinant Pana g 1 and Que ac 1 (20.164 kDa) was evaluated using sera from PFAS patients. Four out of 5 tested sera showed IgE binding to recombinant Pana g 1 (Supplementary figure); however, reactivity to Que ac 1 was consistently stronger (Fig. 1E). Competitive inhibition assays confirmed significant cross-reactivity between Pana g 1 and Que ac 1. Notably, Que ac 1 almost completely inhibited IgE binding to Pana g 1, whereas Pana g 1 failed to inhibit IgE binding to Que ac 1 (Fig. 1F and G).

### Quantification of Pana g 1 in ginseng extract by MRM mass spectrometry and two-site ELISA

Three of the 5 selected target peptides exhibited excellent linearity in the MRM assay (r^2^ > 0.99). Among them, the peptide (AFPQAIK) demonstrated the most reliable quantification, with an LOD of 6.768 ng/mL and an LOQ of 20.304 ng/mL. The concentration of this peptide in the ginseng extract was determined to be 181.67 ng/mL, corresponding to 4.54 μg/mg of the Pana g 1 protein.

**Fig. 3:**
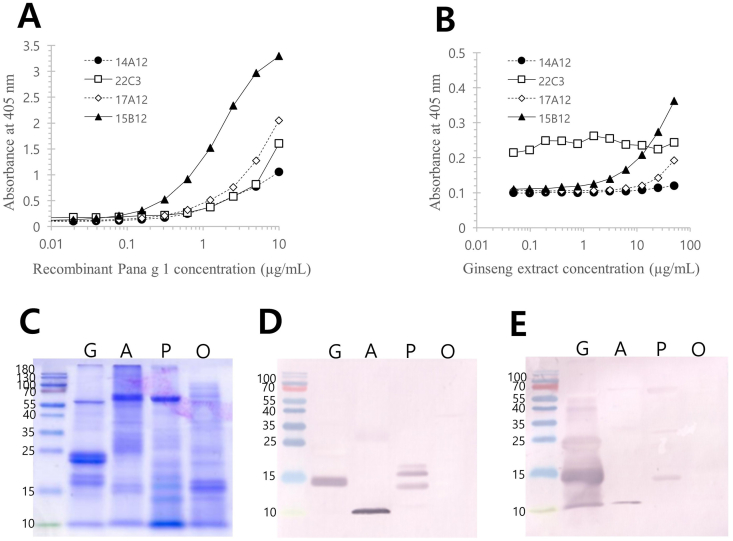
Quantification of Pana g 1 in the extract by a two-site ELISA and specificity of monoclonal antibodies raised against recombinant Pana g 1. Concentrations of recombinant Pana g 1 (A) and natural Pana g 1 in the extract (B) were measured by various combinations of 4 monoclonal antibodies as coating antibodies and 22C3 as a detection antibody. SDS-PAGE and immunoblot analyses were performed to assess cross-recognition of Pana g 1 homologous allergens from peanut, apple, and oak (C) by coating antibody 15B12 (D) and detection antibody 22C3 (E). G: ginseng; A: apple; P: peanut; O: oak.

For immunoassay-based quantification, a two-site ELISA was established using mAbs raised against recombinant protein. The optimal combination was achieved with 15B12 as the capture antibody and biotinylated 22C3 as the detection antibody. The assay demonstrated detection limits, and the extract was 11.04 ng/mL for Pana g 1 and 400 ng/mL for ginseng extract, respectively. Based on this ELISA, the concentration of Pana g 1 in the ginseng extract was 4.3 μg/mg, representing 93.8% of the value obtained by the MRM method. Immunoblotting further confirmed the specificity of these mAbs for recombinant Pana g 1 (Fig. 3).

Interestingly, both coating and detection antibodies also recognize PR-10 homologues from apple (Mal d 1) and peanut (Ara h 8). However, not Que ac 1 from sawtooth oak pollen. This suggests that the ELISA system may be broadly applicable for detecting PFAS-associated PR-10 allergens in fruits and vegetables. Notably, while Mal d 1 appeared partially degraded in apple extract, multiple bands corresponding to ∼17 kDa proteins were detected in peanut extract by 15B12, likely reflecting isoforms or degradation products.

### Pana g 1 content in commercial ginseng beverages

Commercial red ginseng extract sticks contained about 1.0–2.5 μg/mL of Pana g 1, corresponding to 10–25 μg/stick (Table 4A, B). Ginseng tea bags contained 0.311 μg/mL (Table 4C), whereas ginseng drinks contained 0.64–0.886 μg/mL (Table 4DE) and red ginseng drinks 0.571–1.267 μg/mL (Table 4FG). Based on these concentrations, ingestion of a single bottle of red ginseng drink could deliver up to 354.68 μg of Pana g 1, while consumption of a bottle of ginseng drink could result in an intake of approximately 106.3 μg.

**Table 4:** Pana g 1 content in the commercial beverages containing ginseng or red ginseng.

Product	Type	Pana g 1 content			Ginseng or red ginseng content (solid content)	Protein content		
		Concentration (μg/mL)	Vol. (mL)	Quantity		Label	Bradford assay (mg/mL)	Pana g 1/protein (μg/mg)
A	Red ginseng extract stick	2.5	10	25.0 μg/stick	NA^a^	0%	1.65	1.5
B	Red ginseng extract stick	1.0	10	10.3 μg/stick	NA	0.30%	1.26	0.8
C	Ginseng tea	0.3	200	3.1 μg/pouch	NA	NA	0.13	2.3
D	Ginseng drink	0.9	120	106.3 μg/bottle	3-year-old raw ginseng root	0%	0.01	90.0
E	Ginseng drink	0.1	100	6.4 μg/bottle	>80 mg/g	<0.5%	0.07	1.4
F	Red ginseng drink	1.3	280	354.7 μg/bottle	>70 mg/g	0.000%	0.01	130.0
G	Red ginseng drink	0.6	50	28.6 μg/pouch	>70 mg/g	0%	0.07	8.6

a NA, information not available

## Discussion

Many regional and indigenous allergens in Asia remain insufficiently characterized, which limits accurate allergy diagnosis and patient care.^15^ Among these ginseng has long been used as both a traditional medicinal ingredient and a widely consumed health supplement. However, allergic reactions to ginseng, particularly, PFAS represents, are increasingly recognized as a significant health concerns following the ingestion of raw ginseng.

In this study, we focused on PFAS associated with Korean ginseng, specifically characterizing Pana g 1, a PR-10 protein. Genome and transcriptome analyses identified several candidate allergenic molecules, including PR-10, profilin, nsLTP, and thaumatin-like protein. Proteomic profiling confirmed Pana g 1 (PR-10) as the principal causative agent of PFAS. Additional IgE-reactive components of approximately 60 kDa were detected by 2D immunoblotting; however, these could not be conclusively identified, possibly due to their low abundance or post-translational modifications. One-dimensional immunoblotting revealed IgE-binding bands at 15, 25, 45, and 60 kDa, which may reflect non-specific reactions with the abundant protein in the ginseng extract, such as ginseng major protein (GMP).^17^ GMP shares homology with a ribonuclease-like storage protein, although it lacks ribonuclease activity.^18^

IgE reactivity was observed at the 17 kDa component of the ginseng extract, and notably, IgE binding to oak pollen extract was significantly greater than to ginseng extract, consistent with cross-reactivity among PR-10 allergens. This finding supports the idea that PR-10 proteins are the major allergens in ginseng. Importantly, no patients in this study experienced anaphylaxis following red ginseng (steamed and dried ginseng) consumption and cases of food-dependent exercise-induced anaphylaxis (FDEIA) triggered by red ginseng health supplements suggest that heat-stable components may also contribute to ginseng's allergenic potential.^19^^,^^20^

Recombinant Pana g 1 was recognized by 4 of the 5 sera tested. Interestingly, IgE reactivity was stronger to Que ac 1 than to Pana g 1, indicating primary sensitization to oak pollen. In inhibition assays, Que ac 1 effectively blocked most IgE reactivity to Pana g 1, whereas Pana g 1 showed little inhibitory effect on IgE reactivity to Que ac 1 (Fig. 1F, G, H, I). This suggests that sensitization likely originates from oak pollen or a closely related pollen allergen. Supporting this hypothesis, ginseng exhibited limited cross-reactivity to apple allergens: sera from apple-allergic patients bound strongly to a 17 kDa component, likely corresponding to Mal d 1.^21^

Poor inhibition of IgE reactivity to Que ac 1 by Pana g 1 suggests that the dominant IgE epitopes of Que ac 1 may not be major contributors to ginseng-related PFAS. Thus, the specific IgE epitopes responsible for ginseng allergy remain to be identified. The sequence identity between Pana g 1 and other PR-10 allergens is relatively low (41.9%–52.5%), whereas the major PR-10 allergens Bet v 1 and Que ac 1 within the birch homologous group exhibit higher sequence identity (50–83%).^22^ Generally, a sequence homology of ∼35% over a sliding window of 80 amino acids is considered predictive of potential cross-reactivity, although actual clinical relevance requires experimental confirmation.^23^

While Que ac 1 comprises only 0.17% of the soluble proteins in sawtooth oak pollen, Bet v 1 makes up a much higher proportion (13.9%) of the soluble proteins in birch pollen.^24^ The development of an ELISA system to quantify Pana g 1 could therefore play an important role in ensuring the safety of ginseng-derived products. Using a two-site ELISA, we unexpectedly detected relatively high concentrations of Pana g 1 in several commonly consumed ginseng preparations, underscoring the potential relevance of this allergen in daily dietary exposure.

Certain ginsenosides in ginseng are heat-sensitive, and their stability may vary among different products. Most red ginseng extracts are manufactured by subjecting raw ginseng to repeated steaming cycles, followed by low-temperature extraction to minimize ginsenoside degradation.^25^ During this process, both the extracts and Pana g 1 become concentrated before lyophilization, yielding solid content that serves as the basis for various ginseng and red ginseng products. In addition, some beverages may contain intact 3-year-old raw ginseng roots, further contributing to potential allergen exposure.

It should be noted that some red ginseng drinks (products E, F, and G in Table 4) were manufactured in facilities that also process peanuts, soy, peaches, walnuts, pine nuts, and apples, raising the possibility of incidental contamination with PR-10 proteins from these allergenic sources. Nevertheless, the incidence of allergic reactions to red ginseng remains extremely low, likely due to partial denaturation of Pana g 1 during processing. The limited cross-reactivity between Pana g 1 and primary sensitizers like Que ac 1 or Bet v 1 may contribute to the relatively high threshold required to trigger allergic reactions following ginseng consumption.

Potential contamination of PR-10 molecules arising from co-manufacturing with other allergenic sources such as peanut, soy, or apple, may be detected by the mAbs raised against recombinant Pana g 1. In our analysis, we quantified 9.46 ng/mL of Ara h 8 from peanut extract and 11.73 ng/mL of Mal d 1 from apple extract using recombinant Pana g 1 as a standard. These finding indicates this two-site ELISA system may detect PR-10 proteins across a range of fruits and vegetables but not in pollens, highlighting both the versatility and limitations of this approach.

Mass spectrometry with multiple reaction monitoring (MRM) offers advantages over ELISA by enabling the detection of allergenic molecules in processed products, even in polymerized or structurally modified forms.^26^^,^^27^ However, MRM has limitations, particularly for allergens like Bet v 1, where conformational IgE-binding epitopes are critical.^28^ Conseqently, it remains uncertain whether heat-denatured Pana g 1 retains sufficient immunologic activity to provoke allergic responses in sensitized individuals.

In conclusion, Pana g 1 is a clinically relevant allergen responsible for PFAS and represents a valuable diagnostic marker for ginseng-related allergic reactions. The two-site ELISA and MRM mass spectrometry assays developed for quantifying Pana g 1 provide complementary tools that may facilitate the standardization of ginseng extracts and support ongoing allergen monitoring and surveillance in both clinical and industrial settings.

## Abbreviations

PFAS, pollen-food allergy syndrome; PR-10, pathogenesis-related 10; nsLTP, non-specific lipid transfer protein; ELISA, Enzyme-linked immunosorbent assay; mAbs, monoclonal antibodies; MS/MS, tandem mass spectrometry; SRA, sequence read archive; SDS-PAGE; sodium dodecyl sulfate-polyacrylamide gel electrophoresis; PBS, phosphate buffered saline; PBST, PBS containing 0.5% Tween 20; NBT, nitro blue tetrazolium; BCIP, 5-bromo-4-chloro-3-indolyl-phosphate; MRM, multiple reaction monitoring; TMB, 3,3′5,5′-tetramethyl-benzidine; IPTG, isopropyl-b-D-thiogalactopyranoside.

## Data availability statement

The data that support the findings of this study are available from the corresponding author upon request.

## Author contributions

KY Jeong performed purification, cloning, and MRM spectrometry. MK Sang and YS Lee analyzed transcriptomes and genomes. H Kim carried out the proteomic analysis. YJ Shin examined IgE reactivity and conducted two-site ELISA. HK Oh, KH Park, JH Lee, and JW Park collected serum samples. KY Jeong designed the study. KY Jeong wrote the manuscript. JW Park critically revised the manuscript.

## Authors’ consent for publication

All authors read and approved the final version of the manuscript and gave final consent for publication in WAO Journal.

## Declaration of Generative AI and AI-assisted technologies in the writing process

Nothing to disclose.

## Funding information

This work was supported by the National Research Foundation of Korea (NRF) grant funded by the Korean government (MSIT) (No. 2022R1A2C100418411).

## Declaration of competing interest

KY Jeong reports a research grant from the National Research Foundation of Korea (NRF) and a consulting fee from Prolagen Ltd. JW Park reports being an unpaid chief technology officer for Prolagen Ltd. The authors (KY Jeong, JW Park, KH Park, and JH Lee) own stocks in Prolagen Ltd. At the same time, KY Jeong, JH Lee, and JW Park also hold shares in Protia Ltd., Patents have been issued for the allergenic molecules Pana g 1 and hybridomas producing monoclonal antibodies. The reported conflict of interest did not influence academic integrity in analyzing data and writing a paper.

## Figures and Tables

**Supplementary Fig. ure:**
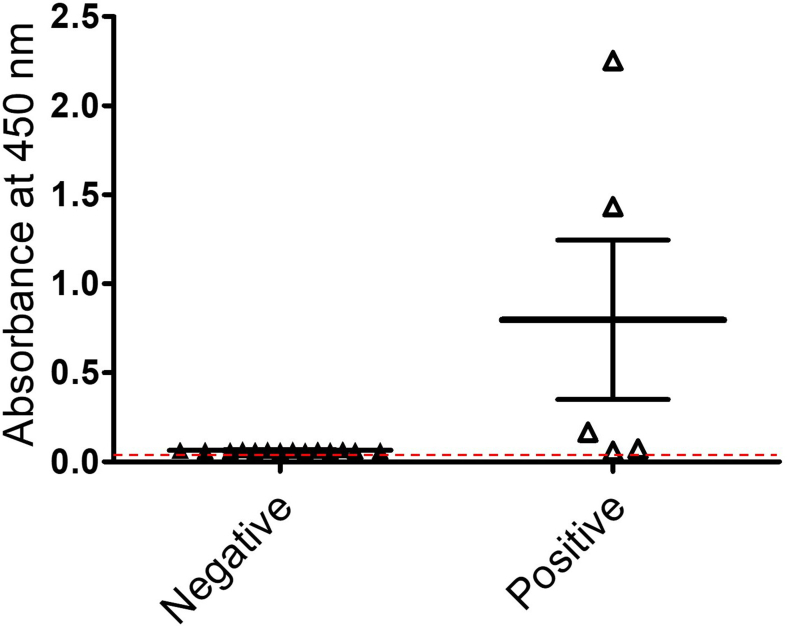
IgE reactivity of recombinant Pana g 1. Red dotted line indicates cutoff value. Bars, mean with SEM
